# Developing effective siRNAs to reduce the expression of key viral genes of COVID-19

**DOI:** 10.7150/ijbs.59151

**Published:** 2021-04-10

**Authors:** Renfei Wu, Kathy Qian Luo

**Affiliations:** Faculty of Health Sciences, University of Macau, Macao SAR, China

**Keywords:** COVID-19, SARS-CoV-2, RNA secondary structure, siRNA, gene silencing

## Abstract

The COVID-19 pandemic has been raging worldwide for more than a year. Many efforts have been made to create vaccines and develop new antiviral drugs to cope with the disease. Here, we propose the application of short interfering RNAs (siRNAs) to degrade the viral genome, thus reducing viral infection. By introducing the concept of the probability of binding efficiency (PBE) and combining the secondary structures of RNA molecules, we designed 11 siRNAs that target the consensus regions of three key viral genes: the spike (S), nucleocapsid (N) and membrane (M) genes of SARS-CoV-2. The silencing efficiencies of the siRNAs were determined in human lung and endothelial cells overexpressing these viral genes. The results suggested that most of the siRNAs could significantly reduce the expression of the viral genes with inhibition rates above 50% in 24 hours. This work not only provides a strategy for designing potentially effective siRNAs against target genes but also validates several potent siRNAs that can be used in the clinical development of preventative medication for COVID-19 in the future.

## Introduction

COVID-19 has infected over 122 million people and caused over 2.7 million human deaths worldwide till March 2021 1. COVID-19 is caused by a positive-sense single-stranded RNA virus named SARS-CoV-2 which has a genome size of approximately 30 kilobases and belongs to the family *Coronaviridae* 2. SARS-CoV-2 uses a spike protein localized on the coat of the viral particle to bind with angiotensin converting enzyme 2 (ACE2) on the membrane of human epithelial cells in the lungs, arteries, heart, kidney, and intestines 3. Upon entry into the cells, the virus will produce RNA-dependent RNA polymerase (RdRP) to synthesize the antisense or negative strand of its genomic RNA for viral replication. In addition, viral RNA can be transcribed into subgenomic RNAs, which will be translated into structural proteins, including spike (S), nucleocapsid (N), and membrane (M) proteins to assemble into more viral particles 4.

One unique feature of RNA viruses is that they use RNA polymerase to synthesize their genetic material, and this enzyme is suggested to have a high error rate during RNA replication 5. This means that when viral RNAs replicate in host cells, new mutated forms of the virus can be generated and subsequently spread to other people. It is estimated that two new mutations can accumulate in the viral genome of SARS-CoV-2 on a monthly basis 6. Some of these mutations may make the new viral strains more contagious and less responsive to the vaccines that were developed based on the original version of the SARS-CoV-2 virus. For example, several more virulent variants of SARS-CoV-2 have been found in the UK, Brazil, and South Africa, and are now spreading to many other countries 7.

As the pace of developing new antiviral drugs seems behind the progression of the COVID-19 pandemic, many people have pinned their hope on vaccination 8,9. However, according to reports, a considerable population is unwilling to be vaccinated, both in developed and developing countries, which will slow the establishment of community immunity 10. In addition, certain susceptible populations have been suggested as inapplicable recipients of the vaccine, including women who are pregnant or within the lactation period and patients with severe basic illnesses or malignant tumors. Thus, developing prompt and flexible antiviral therapies will contribute to the elimination of the COVID-19 pandemic.

An effective way to eliminate RNA-based viruses is to degrade their genetic material by using RNA interference (RNAi), especially small interfering RNAs (siRNAs) or short hairpin RNAs (shRNAs) 11-13. RNAi-mediated gene silencing occurs when double-stranded siRNA molecules enter cells and are recognized by an RNA-induced silencing complex (RISC) that uses one strand of the siRNA to bind to the complementary strand of the target RNA via base pairing. Afterwards, the RISC uses its RNase activity to cleave the target RNA 14. RNAi technology has been widely used to silence gene expression in human cells in the last twenty years 15-18. The past several years have witnessed the rapid growth of RNAi-based clinical trials for viral infections such as Epstein-Barr virus (EBV) 19, hepatitis B virus (HBV) 20, human immunodeficiency virus (HIV) 21, SARS-CoV 22 and the raging SARS-CoV-2 13.

One of the challenges of using RNAi to reduce gene expression is that the length of an siRNA is very short, containing only 19-23 nucleotides, while the length of the target RNA strand is much longer, ranging from several hundred to thousands of nucleotides long. Thus, it is difficult to choose target sites on the RNA to design siRNAs. Due to the complexity of intracellular RNAi processing, as well as the dynamic conformation of target RNA molecules in different physiological statuses, the existing siRNA design tools can only partially predict plausible targets and still requires extensive validation to achieve better efficacy in a wet lab 23. In this study, we developed a method to design effective siRNAs against three viral genes of SARS-CoV-2 and tested their efficacies in reducing viral gene expression in human cells.

## Materials and methods

### Cell culture

Human umbilical vascular endothelial cells (HUVECs) and human lung carcinoma A549 cells were purchased from the American Type Culture Collection (ATCC, Rockville, MD, USA). All cells were cultured in DMEM (#12100046, Thermo Fisher Scientific, USA) supplemented with 10% fetal bovine serum (#10270106, Gibco, USA) and 1% penicillin-streptomycin antibiotics (#15140122, Thermo Fisher Scientific, USA). All cells tested negative for mycoplasma contamination.

### Transfection of plasmid and siRNA

An overexpression plasmid for a mutant variant of the S (D614G) gene (#GS-200519_A012, pCDNA3.1(+)-S-D614G) was purchased from Genewiz (Suzhou, China); plasmids of the N (#HH-gene-158, pCDNA3.1(+)-2019-nCoV-N) and M (#HH-gene-135, pCDNA3.1(+)-2019-nCoV-M) genes were purchased from HedgehogBio (Shanghai, China). siRNAs were purchased from GeneralBio (Chuzhou, China). For transfection of plasmids and siRNAs, 1×10^5^ cells were seeded into each well of a 12-well plate and grown overnight. Afterwards, 30 ng of plasmid DNA and 10 pmol of siRNA (at a final concentration of 10 nM) were added to 800 µL Opti-MEM™ (#22600050, Gibco, USA). Then, 1.6 µL Lipofectamine™ 2000 (#11668019, Invitrogen, Waltham, MA, USA) was added to the plasmid:siRNA solution and the mixture was incubated at room temperature for 30 min. Then, the mixture was added to the cells and incubated for 6 hours at 37ºC. After 18 hours of incubation, cells were harvested for RNA isolation and qPCR measurement.

### Real-time quantitative polymerase chain reaction (qPCR)

RNA was extracted with TRIzol (#15596026, Thermo Fisher Scientific, USA) and isopropanol precipitation methods. cDNA was synthesized using an iScript™ cDNA Synthesis Kit (#1778890, Bio-Rad, USA), and qPCR was performed using iTaq™ Universal SYBR® Green (#1725122, Bio-Rad, USA) and analyzed with an Applied Biosystems™ 7500 Fast Real-Time PCR Detection System (Thermo Fisher Scientific, USA). The changes in mRNA levels were calculated using the ΔΔCt method with the mRNA level of GAPDH as an internal control. The used primers are listed in Table 1.

### Statistical analysis

Experiments were repeated at least four times, and the data were analyzed using one-way ANOVA as processed by GraphPad Prism 8 software (San Diego, CA, USA). All the analyzed results are presented as the mean ± SD (standard deviation). Statistical significance is shown as *p < 0.05, **p < 0.01, ***p < 0.001, ****p < 0.0001; ns, not significant.

## Results

### Selection of nine variants from the major prevalent viral strains worldwide

**Figure 1:**
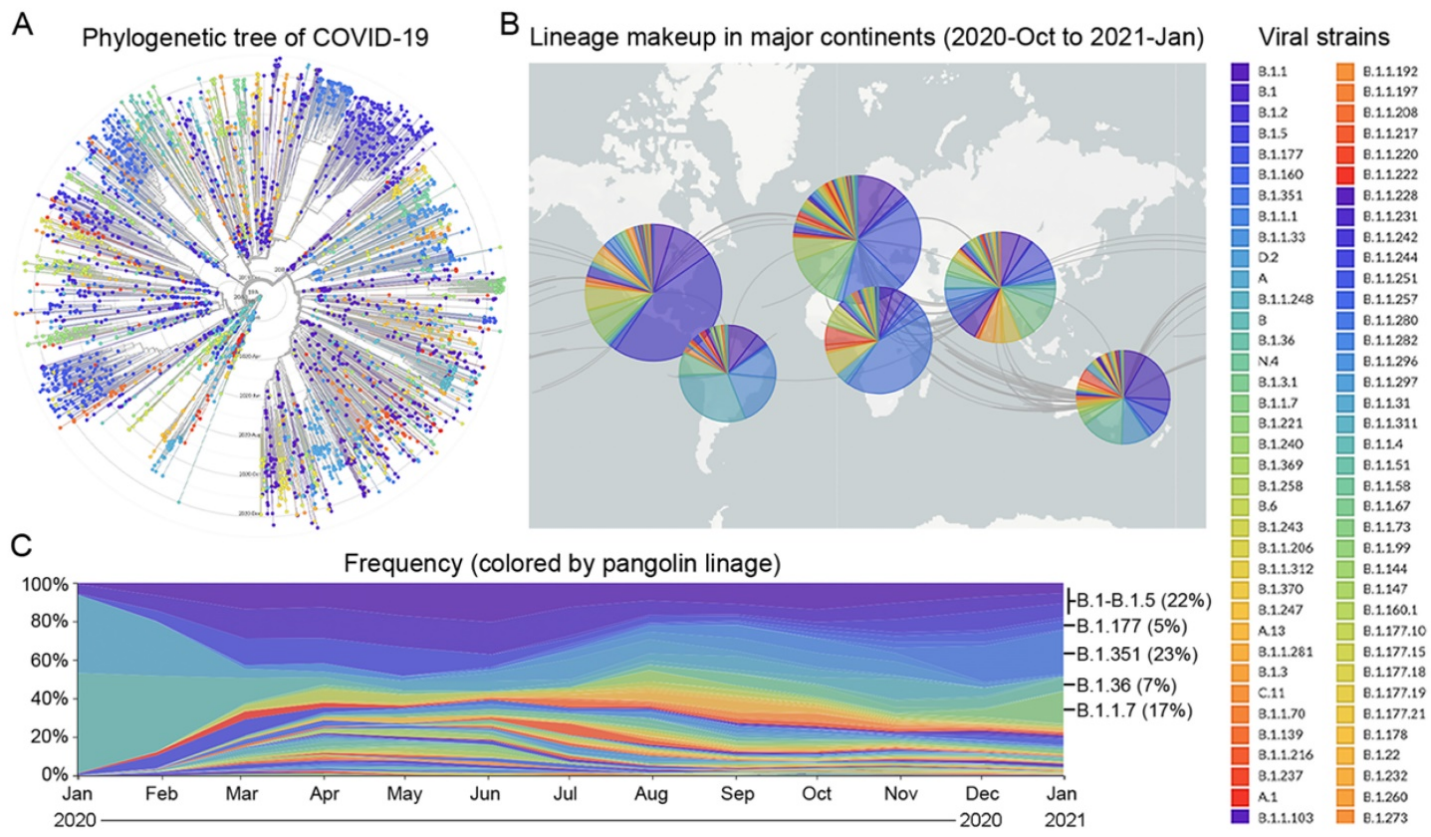
** Prevalence of major viral strains of COVID-19 in 2020 and early 2021. A.** The phylogenetic tree of COVID-19 from 2019-Dec to 2021-Jan plotted by the GISAID platform. Each viral strain was colored based on the pangolin lineage. **B.** The geographic distribution of major viral strains between 2020-Oct and 2021-Jan. **C.** The evolution of COVID-19 from 2020-Jan to 2021-Jan based on the frequency of major viral strains that were sequenced chronologically.

Since the beginning of the COVID-19 pandemic in January 2020, many variants of the SARS-CoV-2 virus have been detected in over 200 countries 24. Based on the times and locations of the viral strains that appeared in the past 12 months, we prepared a phylogenetic tree and a pangolin lineage of COVID-19 from the GISAID platform using Nextstrain open source project 25,26, which showed that this virus has quickly mutated and spread across the five continents (Figure 1A and B). According to the frequency of viral mutations and the trend of prevalence between late 2020 and early 2021, nine strains were selected, which account for 74% of the total prevalent strains in the world (Supplementary Table 1). Six strains, B.1 (5%), B.1.1 (5%), B.1.177 (5%), B.1.1.50 (3%), B.1.2 (7%), and B.1.5 (2%), are prevalent in the Americas, giving a total percentage of 27%. For the other three strains, B.1.351 (23%) is prevalent in Africa, B.1.1.7 (17%) is prevalent in Europe, especially in the UK, and B.1.36 (7%) is prevalent in Asia (Figure 1C).

### Calculation of the probability of binding efficiency (PBE) based on single-stranded information of RNA molecules

Although RNA is synthesized into a single strand, its complementary nucleotides, such as A=U and G≡C, can bind to each other via hydrogen bonds to produce different secondary structures, such as loops, stems, and hairpins. Previously, we reported that nucleotides located in the loop structure are in a single-stranded configuration and are easier for siRNAs to target, while nucleotides located in the stem region are in a double-stranded configuration and are harder for siRNA to target 16. As the binding between siRNA and the target RNA is crucial for RISC to degrade target RNA, these single-strand loop regions can be selected as potential sites for designing siRNAs.

**Figure 2:**
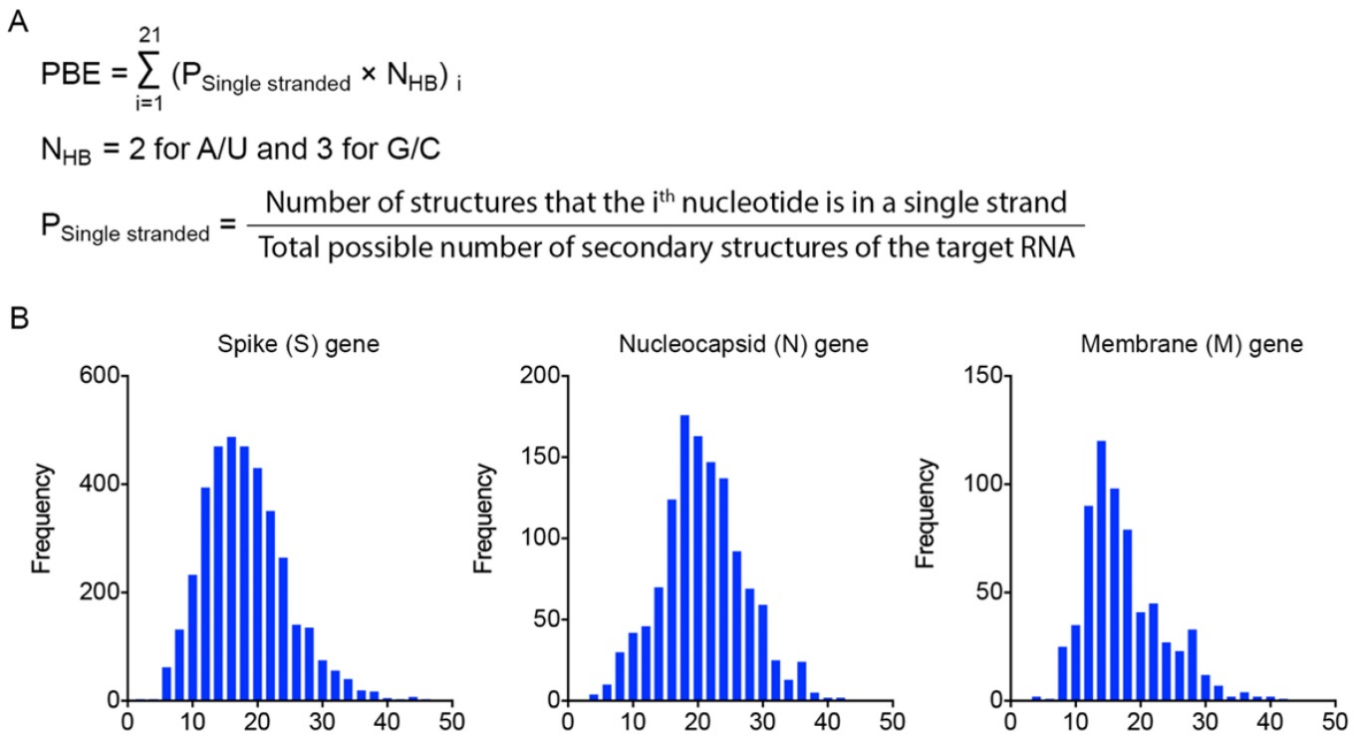
** Calculation of the probability of binding efficiency (PBE) based on single-stranded information of RNA molecules. A.** PBE was calculated by summing the hydrogen bonds formed between siRNA and the target region of RNA molecules. N_HB_ refers to the number of hydrogen bonds formed by each nucleotide base pair. **B.** The distribution of PBE values for three viral genes: spike (S), nucleocapsid (N) and membrane (M) genes.

To identify effective target sites on the RNA molecule, we designed a formula to calculate the probability of binding efficiency (PBE) between an siRNA and its target RNA based on the single-strand information obtained from the Mfold web server 27. PBE calculates the total number of hydrogen bonds between 21 nucleotides of an siRNA and the complementary region of the target RNA based on all the predicted secondary structures (Figure 2A).

We then calculated the PBE values of all possible siRNA candidates against the spike (S), nucleocapsid (N) and membrane (M) viral genes. The distribution curves show that the PBE values of most of the siRNA candidates are below 30, while the PBE values of a small portion of the siRNA candidates are above 30 (Figure 2B). It should be emphasized that for different genes with different length and the number of secondary structures, the values of PBE can differ significantly. Thus, there is no universal cut-off to indicate the efficacy of siRNAs. We recommend choosing the ones with the highest PBE values as candidates. We then selected the 3-4 top-ranked siRNAs with the highest PBE values from individual viral genes for structural and functional assessments.

### siRNAs with high PBE values can effectively reduce the expression of the spike gene

**Figure 3:**
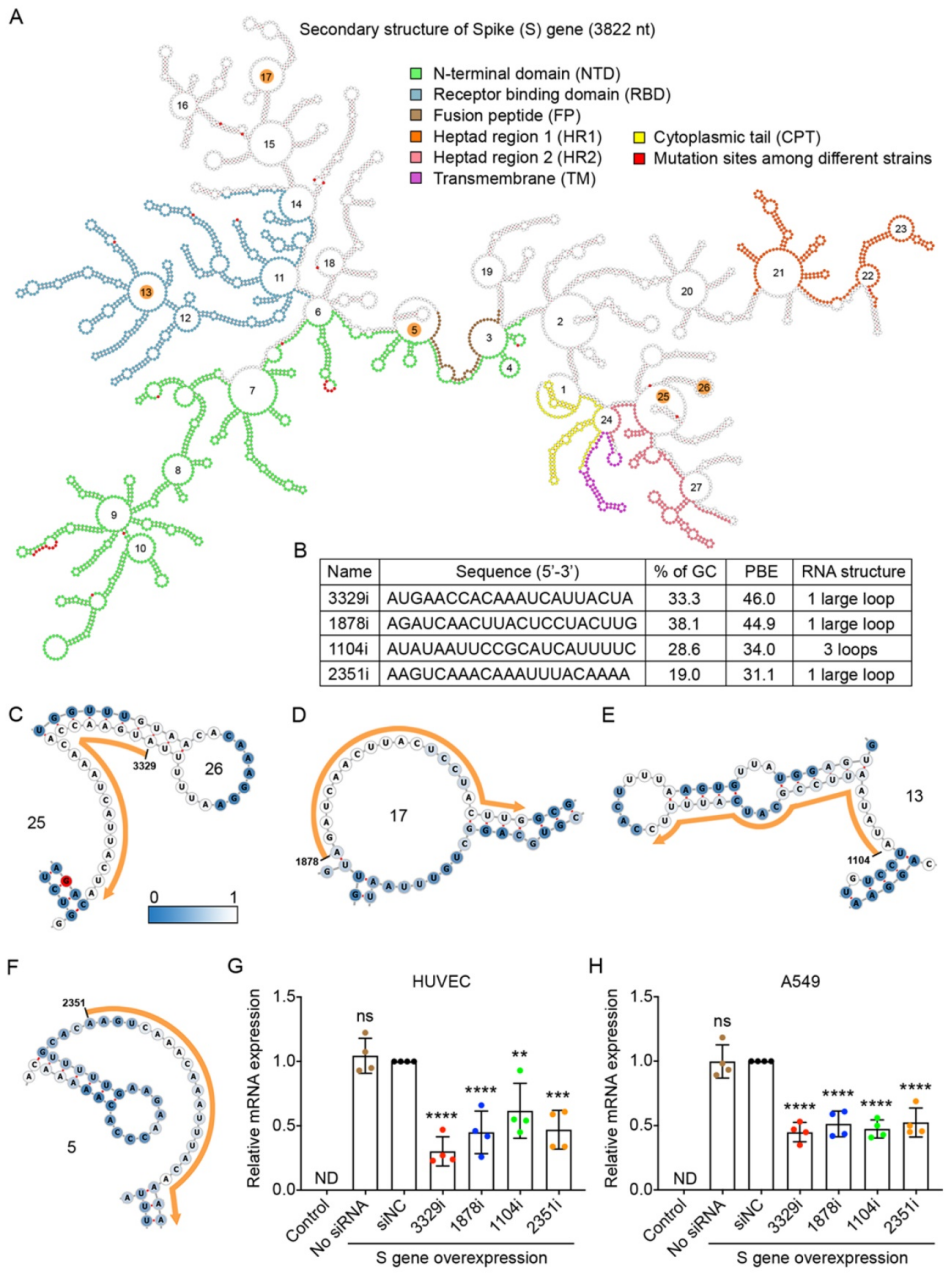
** Selection of high PBE siRNAs for the spike (S) gene. A.** The secondary structure of the RNA molecule of the S gene. The nucleotide sequences corresponding to functional protein domains are labeled in different colors. Mutated sites among the nine viral strains are labeled in red. The loops numbered 5, 13, 17, 25 and 26 are highlighted in orange, as these regions were selected for siRNA targeting. **B.** The sequence, GC content, PBE and RNA structure of the four selected siRNAs. **C-F.** Detailed structures of targeted regions by four siRNAs, 3329i (C), 1878i (D), 1104i (E) and 2351i (F), on the S gene with the siRNA sequence outlined in orange. The color of each nucleotide reflects the possibility of being single-stranded. **G-H.** Real-time qPCR was used to measure the relative expression of the S gene in both HUVECs (G) and A549 cells (H) after transfection with the plasmid DNA of the S gene and individual siRNAs at 24 hours. Cells that were not transfected were used as a background control. All values were normalized to the siNC group, in which cells were transfected with the S gene and a negative control siRNA simultaneously. In No siRNA group, only plasmid DNA was transfected.

We used the RNA sequence of the S gene from the B.1 strain, as this strain is currently the origin of the most prevalent strains in the world 24. The S gene of the B.1 strain contains a single mutation from A to G at nucleotide 1841, resulting in a change from aspartic acid to glycine at amino acid position 614 28. The RNA sequence of the S gene was folded by using the RNAfold web server 29, and its secondary structure was displayed by using the Forna platform 30 (Figure 3A). We also compared the sequences of the S gene between the original B.1 strain and the other eight variants and found that there were 17 mutations, most of which could cause changes to the amino acid sequence. These 17 mutation sites are highlighted in red in Figure 3A.

In theory, as the RNA sequence of the S gene contains 3,822 nucleotides (nt), 3,802 siRNA candidates with a length of 21 nt can be generated. We calculated the PBE values of all siRNA candidates against the S gene and found that they ranged from 2.3 to 46.0. The top four candidate siRNAs with the highest PBE values of 31.1-46.0 (Figure 3B) and outside of the 17 mutation sites were selected (Figure 3A).

In the detailed secondary structures of these siRNAs, each nucleotide is colored from steel blue to white, which indicates an increased probability of hydrogen bond formation between the siRNA and target RNA (Figure 3C-F). Among these siRNAs, 3329i and 1878i had higher PBE values, and their targeting regions did not encode any functional domains of the S protein (Figure 3A-D). The siRNA 1104i targets loop 13, which encodes a small region of the receptor binding domain (RBD) (Figure 3A and E). The siRNA 2351i targets loop 5, which partially encodes the fusion peptide (FP) of the S protein (Figure 3A and F).

To determine the gene silencing effect of the siRNA candidates against the S gene, we first showed that this viral gene was exogenously expressed in human HUVECs and A549 cells at the level comparable to the high expression of a housekeeping gene (GAPDH). The qPCR results showed that the expression of S gene was not detectable (ND) in these two types of cells without transfecting the viral gene (Figure 3G and H).

To test the silencing efficiency of the designed siRNAs, plasmid DNA expressing the S gene and individual siRNAs (10 nM) were transfected into HUVECs and A549 cells using Lipofectamine 2000 for 6 hours. The cells were then incubated for 18 hours before RNA isolation and quantification. The results showed that although the S gene was expressed at very high levels in the cells, all four siRNAs significantly reduced its mRNA levels in both HUVECs and A549 cells (Figure 3G and H). Specifically, in HUVECs, the 3329i siRNA, which had the highest PBE value, produced the greatest level of gene silencing of 70%, while the 1878i and 2351i siRNAs reduced the levels of S gene expression by over 50% (Figure 3G). In A549 cells, although siRNA 3329i reduced S gene expression by 55%, the other three siRNAs achieved similar silencing effects ranging from 48-53% (Figure 3H). The results suggest that the PBE value is useful to select candidate regions within RNA for designing siRNAs.

### Designed siRNAs can effectively reduce the expression of the nucleocapsid gene

**Figure 4:**
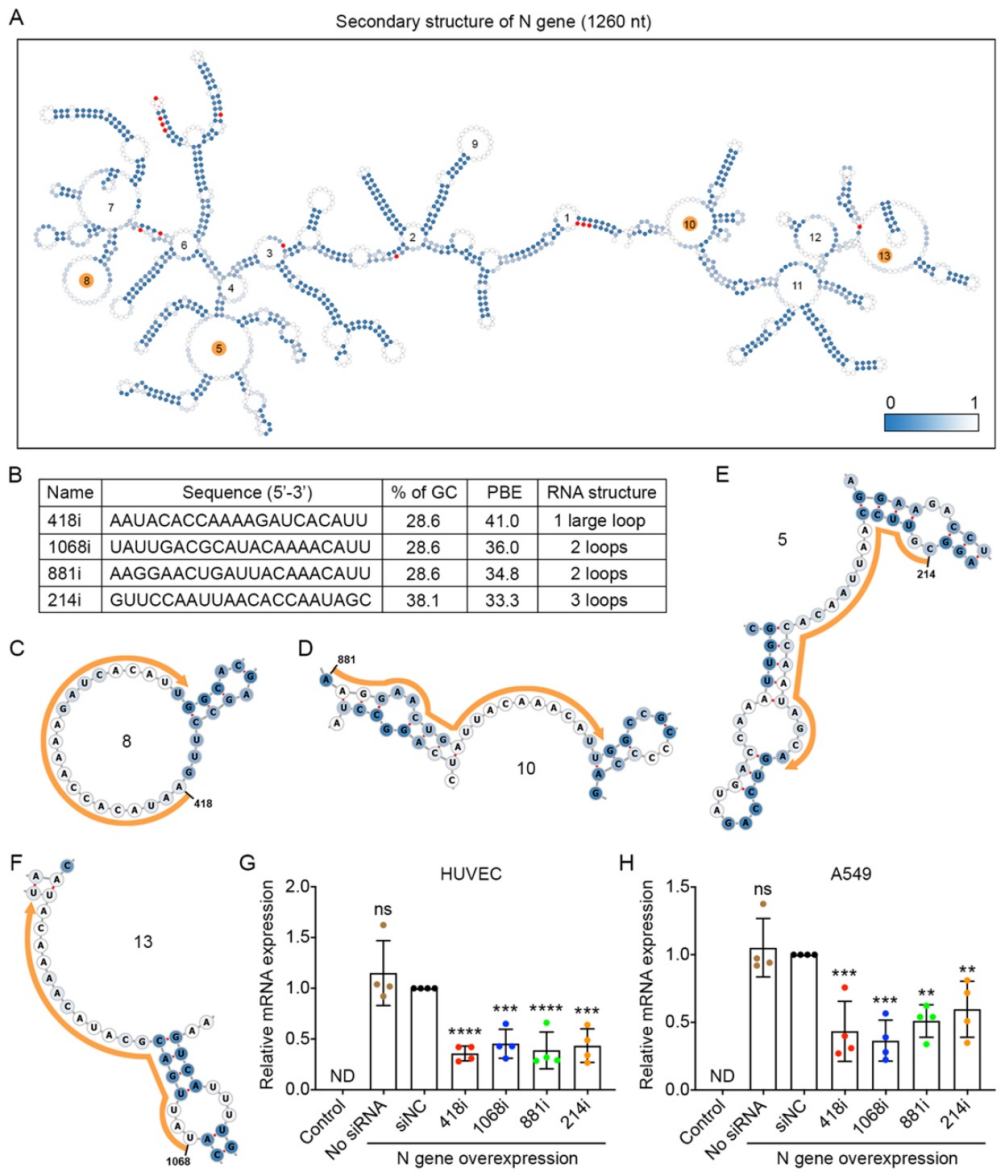
** Selection of high PBE siRNAs for the nucleocapsid (N) gene. A.** The secondary structure of the RNA molecule of the N gene where the color of each nucleotide reflects the possibility of being single-stranded. The mutated sites among the nine viral strains are labeled in red. The loops numbered 5, 8, 10 and 13 are highlighted in orange, as these regions were selected for siRNA targeting. **B.** The sequence, GC content, PBE and RNA structure of the four selected siRNAs. **C-F.** Detailed structures of targeted regions by four siRNAs, 418i (C), 881i (D), 214i (E) and 1068i (F), on the N gene where the siRNA sequences are outlined in orange. **G-H.** Real-time qPCR was used to measure the relative expression of the N gene in both HUVECs (G) and A549 cells (H) after transfection with the plasmid DNA of the N gene and individual siRNAs at 24 hours. Cells that were not transfected were used as a background control. All values were normalized to the siNC group, in which cells were transfected with the N gene and a negative control siRNA simultaneously.

The nucleocapsid (N) gene is conserved among coronaviruses, and the encoded protein is important for viral replication and eliciting the immune response for SARS-CoV-2 31,32; thus, inhibiting N gene amplification is a good strategy to prevent viral propagation. We compared the sequences of the N gene among the nine prevalent viral strains and found 13 mutations. The locations of these mutated nucleotides are indicated in red in the secondary structure of the N gene with 1,260 nucleotides (Figure 4A). We then chose the top four siRNAs that had the highest PBE values of 33.3-41.0 among the possible 1,240 siRNA candidates (Figure 4B-F) and compared their silencing effects in the two cell lines that overexpressed the N gene (Figure 4G and H). In HUVECs, 418i siRNA, which had the highest PBE value, produced the best silencing effect, with a reduction of N gene expression at mRNA level of 64%, and the other three siRNAs generated slightly lower but significant silencing effects of 55-61% (Figure 4G). In A549 cells, the top two ranked siRNAs in terms of PBE values, 418i and 1068i, achieved a better silencing effect than the other two siRNAs (Figure 4H). Taken together, the top three ranked siRNAs reduced N gene expression by over 50% in both types of cells.

### Designed siRNAs can effectively reduce the expression of the membrane gene

**Figure 5:**
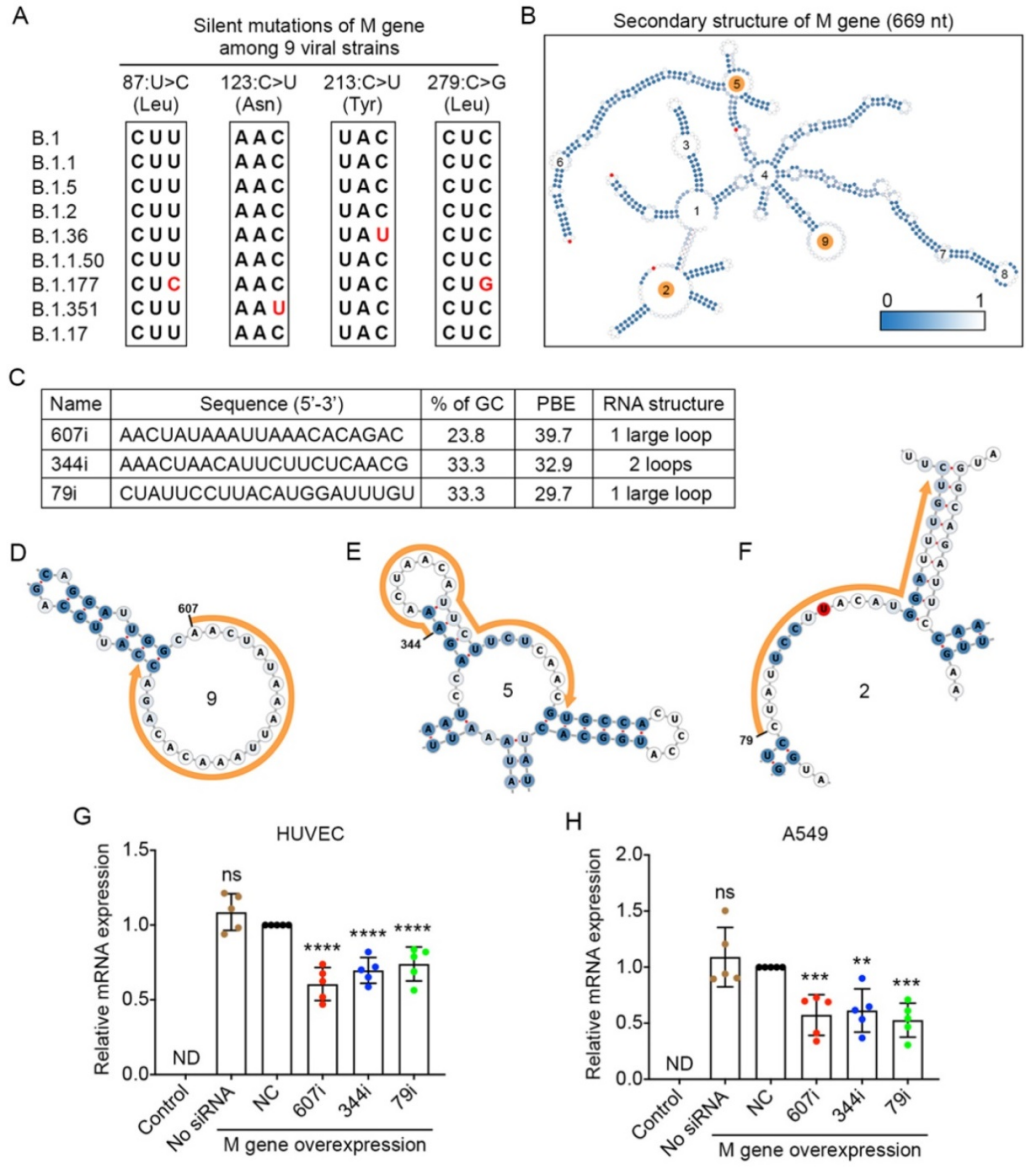
** Selection of high PBE siRNAs for the membrane (M) gene. A.** Mutation information of the M gene among the nine viral strains. **B.** The secondary structure of the RNA molecule of the M gene where the mutated sites are labeled in red. The loops numbered 2, 5 and 9 are highlighted in orange, as these regions were selected for siRNA targeting. **C.** The sequence, GC content, PBE and RNA structure of the three selected siRNAs. **D-F.** Detailed structures of targeted regions by three siRNAs, 607i (D), 344i (E) and 79i (F), on the M gene with the siRNA sequences outlined in orange. **G-H.** Real-time qPCR was used to measure the relative expression of the M gene in both HUVECs (G) and A549 cells (H) after transfection with the plasmid DNA of the M gene and individual siRNAs at 24 hours. Cells that were not transfected were used as a background control. All values were normalized to the siNC group in which cells were transfected with the M gene and a negative control siRNA simultaneously.

The membrane (M) gene encodes the M glycoprotein that is located at the membrane of the viral particle. It is the most abundant structural protein of the SARS-CoV-2 virus 33. By comparing the viral sequences of the M gene among the nine strains, we found that only four point mutations occurred in three of the viral strains. None of these mutations caused any changes at the amino acid level (Figure 5A).

The secondary structure of the M gene was generated, and ss-count information was used to calculate the PBE values covering the entire length of M gene with 669 nucleotides (Figure 5B). Based on the PBE values of 649 candidates, we selected the top three siRNAs (Figure 5C), and the detailed secondary structures of these siRNA-targeted regions are shown in Figure 5D-F. The results showed that both types of cells expressed high levels of the M gene after the transfection of plasmid DNA for the M gene (Figure 5G and H). However, when molecules of siRNA 607i were transfected together with the plasmid DNA of the M gene, they reduced M gene expression by 39-43% in both HUVECs and A549 cells (Figure 5G and H). The other two siRNAs reduced M gene expression by 39-47% in A549 cells but only decreased M gene expression by 26-30% in HUVECs (Figure 5G and H).

## Discussion

The S and N proteins are the major focuses for developing antiviral drugs and vaccines for SARS-CoV-2 31,35, especially the D614G form of the S protein, which has been suggested to enhance viral fitness and has become prevalent in major viral strains 28. In this study, we used siRNA to decrease the levels of viral RNAs, which has several advantages over other methods.

First, siRNAs directly exert silencing effects and act faster than other types of RNAi, such as shRNA or microRNA (miRNA), as the latter two need to be intracellularly expressed and processed into mature forms before they can bind with the target genes.

Second, the design of siRNAs is flexible for highly mutable targets such as the RBD of the S gene 36. In this study, we avoided mutated regions identified among the nine prevalent strains. In the future, when more mutations emerge in the viral genome, we can modify our siRNAs to target these new viral strains. Furthermore, we can introduce a pool of siRNAs to target multiple variants at the same time. In this manner, the development of siRNA-based preventative or curative medication can readily keep pace with the evolution of SARS-CoV-2.

Third, as a noninvasive approach, inhalation of liposome-encapsulated siRNAs can theoretically reduce the risk of nasal infection to SARS-CoV-2 without bringing about severe side effects 37. In addition, such an approach more easily reaches a relatively high drug concentration in the cells lining the respiratory tract, and an overall lower drug dosage is required to achieve a protective purpose.

Although there has been no consensus on how many viral particles are needed for an effective infection, it is widely observed that a higher viral load positively correlates with a higher risk of transmission 38 and increased disease severity 39. A cohort study reported that in the respiratory tract of patients with severe COVID-19 there were approximately 7×10^6^ viral copies per milliliter of specimens 40. In our study, we transfected cells with 30 ng of plasmid DNA corresponding to 3.3-4.9×10^9^ DNA molecules per milliliter of culture medium in a culture well containing 1×10^5^ cells; thus, more than 1×10^4^ DNA molecules were estimated to enter each transfected cell. Under the cytomegalovirus promoter known to have a strong transcriptional effect, many more RNA molecules were synthesized, which resulted in a high abundance of expressed viral genes with a threshold cycle (Ct) number comparable to that of a highly expressed housing keeping gene, GAPDH, in the host cells. Even at such high concentrations of viral genes, the siRNAs selected based on high PBE values significantly reduced the expression of the viral genes within 24 hours. From this point of view, we expect our siRNAs to produce better effects when applied clinically, as the RNA copy number is usually lower in real infections 41.

In summary, we used the PBE value and the secondary structure of three key viral genes to guide the design of candidate siRNAs. By using an *in vitro* viral expression and targeting model, we showed that most of the selected siRNAs achieved a silencing effect of over 50%. In response to the progression of the COVID-19 pandemic and the constant evolution of SARS-CoV-2, we proposed a flexible and promising siRNA-based approach for viral prevention as well as early treatment. For the application prospects in the future, this approach can complement the preventative medication for populations that are unrecommended for vaccination, as well as persons who need a quick and reinforced prevention to viral infection.

## Supplementary Material

Supplementary table.

## Figures and Tables

**Table 1:** Primer list.

Target gene	Direction	Primer (5' - 3')
Spike (S) gene	Forward	GCTGGTGCTGCAGCTTATTA
	Reverse	AGGGTCAAGTGCACAGTCTA
Nucleocapsid (N) gene	Forward	GGGGAACTTCTCCTGCTAGAAT
	Reverse	CAGACATTTTGCTCTCAAGCTG
Membrane (M) gene	Forward	TGTGACATCAAGGACCTGCC
	Reverse	CTGAGTCACCTGCTACACGC
GAPDH gene	Forward	CTGGGCTACACTGAGCACC
	Reverse	AAGTGGTCGTTGAGGGCAATG
