# Supplementary material for: Developing effective siRNAs to reduce the expression of key viral genes of COVID-19
Source: Int J Biol Sci. 2021 Apr 10;17(6):1521–9. doi: 10.7150/ijbs.59151 (PMC8071776; doi:10.7150/ijbs.59151)
Supplement: Supplementary file 1 — Supplementary table. [file ijbsv17p1521s1.pdf]

Supplementary Table 1. Information of the nine viral strains used in this study.

Lineage	Virus name	Accession No.	Collected	Originating lab	Submitting lab	Authors
B.1	hCoV-19/Germany/ BY-ChVir-1483/2020	EPI_ISL_450208	8/2/2020	Department of Virology, Charite - Universitaetsmedizin Berlin	Department of Virology, Charite - Universitaetsmedizin Berlin	Boehmer,M.M., Buchholz,U., Corman,V.M., Hoch,M., Katz,K., Marosevic,D.V., Boehm,S., Woudenberg,T., Ackermann,N., Konrad,R., Eberle,U., Treis,B., Dangel,A., Bengs,K., Fingerle,V., Berger,A., Hoermansdorfer,S., Ippisch,S., Wicklein,B., Grahl,A., Poertner,K., Walter,M., Protzer,U., Liebl,B., Haas,W., Sing,A., Drosten,C., Zapf,A., Jones,T.C. Heiden,M., Rexroth,U., Hamouda,O., Schneider,J., Veith,T., Muehlemann,B., Woelfel,R., Antwerpen,M., Muller,N., Zeitlmann,N., Boender,T.S., Cai,W., Reich,A., an der
B.1.1	hCoV-19/Ireland/KY-NVRL-20G35628 /2020	EPI_ISL_605797	20/2/2020	National Virus Reference Laboratory, University College Dublin	Irish Coronavirus Sequencing Consortium - Helixworks	Sachin Chalapati, Conor Crosbie, Nimesh Pinnamaneni
B.1.5	hCoV-19/Brazil/SP-01/2020	EPI_ISL_412964	25/2/2020	Hospital Israelita Albert Einstein	Instituto Adolfo Lutz Interdisciplinary Procedures Center Strategic Laboratory	Jaqueline Goes de Jesus, Claudio Tavares Sacchi, Daniela Bernardes Borges da Silva, Ingra Morales Claro, Flávia Cristina da Silva Sales, Claudia Regina Gonçalves, Joshua Quick, Maria do Carmo, Sampaio Tavares Timenetsky, Nicholas James Loman, Andrew Rambaut, Ester Cerdeira Sabino, Nuno Rodrigues Faria
B.1.2	hCoV-19/USA/UT-QDX-2632/2020	EPI_ISL_604974	12/3/2020	Quest Diagnostics	Quest Diagnostics	Rosenthal,S.H., Gerasimova,A., Kagan,R.M., Anderson, B., Grover, D., Livingston, K.E., Hua, M., Liu Y., Shalhout, D.F., Owen, R., Lacbawan, F.

**Lineage**	**Virus name**	**Accession No.**	**Collected**	**Originating lab**	**Submitting lab**	**Authors**
B.1.36	hCoV-19/Saudi Arabia/4637/2020	EPI_ISL_469241	15/3/2020	Special Infectious Agents Unit, King Abdulaziz University	Special Infectious Agents Unit, King Abdulaziz University	Azhar,E.I., Hassan,A.M., Tolah,A.M., Uthman,N.A., Al-Sobahy,T.L., Farraj,S.A., El-Kafrawy,S.A.
B.1.1.50	hCoV-19 /Palestine/86/2020	EPI_ISL_596559	22/6/2020	Palestinian Ministry of Health	Molecular Genetics Lab, Arab American University	Nouar Qutob, Zaidoun Salah, Damien Richard, Hisham Darwish, Husam Sallam, Issa Shtayeh, Osama Najjar, Mahmoud Ruzayqat, Dana Najjar, Francois Balloux, Lucy van Dorp
B.1.177	hCoV-19/Tunisia/ 19695/2020	EPI_ISL_733500	12/7/2020	1-Laboratory of Microbiology, National Reference Lab, Charles Nicolle Hospital; 2-University of Tunis ElManar, Faculty of Medicine of Tunis	1-Clinical and Experimental Pharmacology Lab, University of Tunis El Manar, Tunisia. 2-Neurodegenerative diseases and psychiatric troubles, Razi Hospital, Tunisia. 3- Ministry of Health, National Observatory of New and Emerging Diseases, Tunisia	Ilhem Boutiba-Ben Boubaker, Sameh Trabelsi, Nissaf Ben Alaya, Maher Kharrat, Alia Ben Kahla, Jalila Ben, Khelil, Salma Abid, Sana Ferjani, Mouna Ben Sassi, Mouna Safer, Guedi Ali Barreh, Habiba Ben Romdhane, Souissi Amira, Sarra Chamman, Hanen El Jebari, Asma Ferjani, Gaies Emna, Riadh Daghfous, Riadh Gouider
B.1.351	hCoV-19/South Africa/NHLS-UCT-GS-0683/2020	EPI_ISL_700450	22/10/2020	Knysna CDC wc WLC	NHLS/UCT, University of Cape Town and National Health Laboratory Service	Arash Iranzadeh, Deelan Doolabh, Lynn Tyers, Bruna Galvao, Innocent Mudau, Marvin Hsiao, Kruger Marais, Diana Hardie, Stephen Korsman, Carolyn Williamson
B.1.1.7	hCoV-19/England/ PORT-2D5284/2020	EPI_ISL_741692	2020	Centre for Enzyme Innovation, University of Portsmouth / Translational Research Laboratory, Portsmouth Hospitals NHS Trust	COVID-19 Genomics UK (COG-UK) Consortium	Angela Beckett,Yann Bourgeois,Garry Scarlett,Sharon Glaysher,Scott Elliott,Kelly Bicknell,Robert Impey,Allyson Lloyd,Sarah Wyllie,Ethan Butcher,Anoop Chauhan,Samuel Robson
